# Assessing the Malaysian Regulatory Process for Medicinal Product Approval: An OpERA Methodology and Standardized Reporting Approach

**DOI:** 10.1007/s43441-025-00846-3

**Published:** 2025-07-25

**Authors:** Noraisyah Mohd Sani, Siti Hidayah Kasbon, Kian Yee Yap, Muhamad Firdaus Abdullah, Rosliza Lajis, Azuana Ramli, Neil McAuslane, Magda Bujar, Adem Kermad

**Affiliations:** 1 National Pharmaceutical Regulatory Agency, Lot 36, Jalan Universiti (Jalan Prof Diraja Ungku Aziz), 46200 Petaling Jaya, Selangor Malaysia; 2 Centre for Innovation in Regulatory Science (CIRS), 70 St Mary Axe, London, EC3A 8BE UK

**Keywords:** National Pharmaceutical Regulatory Agency (NPRA), Optimising Efficiencies in Regulatory Agencies (OpERA), Regulatory strengthening, Benchmarking

## Abstract

**Background:**

The Malaysian National Pharmaceutical Regulatory Agency (NPRA) has partnered with the Centre for Innovation in Regulatory Science (CIRS) since 2018 to analyze the approval processes for new active substances (NASs) and biosimilars. Findings from the study on approvals in the year 2017 led to the introduction of several improvement strategies. This study provides an overview of the current review process, including registration pathways, and compares approval times for NASs approved in 2019 vs 2017 to evaluate the impact of the improvement strategies.

**Methods:**

NPRA representatives completed the Country Report using CIRS’ Optimizing Efficiencies in Regulatory Agencies (OpERA) questionnaire, identifying key milestones, target timelines, good review and quality decision-making practices, and provided metrics on 24 NASs approved in 2019.

**Results:**

Most indicators for good review practices were implemented by NPRA, with guidelines, standard operating procedures, review templates, and several identifiable quality decision-making practices being in place. NPRA has also introduced several registration pathways with the aim of accelerating approval timelines. Median total approval time decreased from 515 calendar days in 2017 to 399 calendar days in 2019. Reductions were also noted in the median time between dossier receipt and initiation of NPRA scientific assessment.

**Conclusions:**

The study indicates that improvement strategies implemented in 2018 favorably reduced approval times, based on a comparison of products approved in the year 2017 and 2019. Ongoing evaluation of regulatory processes and performance is crucial to identify areas for improvement, eliminating unnecessary steps, and enabling a streamlined and efficient approach.

**Supplementary Information:**

The online version contains supplementary material available at 10.1007/s43441-025-00846-3.

## Introduction

Regulatory agencies continually seek to strengthen their review of new medicines in order to fulfil their mandate to protect and promote public health by enabling access to good-quality, safe, and effective medicines in a timely manner [1]. To date, regulatory agencies worldwide, including the Malaysian National Pharmaceutical Regulatory Agency (NPRA), face pressure to accelerate the review process for market approval. The review process is expected to be competent, flexible, risk-based, and efficient, given lengthy review procedures might delay access to life-saving drugs [2]. NPRA actively considers methods for improvements that can benefit both the agency and applicants. This involves exploring methods to accelerate evaluation while maintaining efficacy, safety, and quality. NPRA also aims to enhance staff efficiency by fostering awareness of regulatory science developments.

NPRA collaborated with the Centre for Innovation in Regulatory Science (CIRS), a neutral, UK-based, research organization, to collect milestone data identifying time periods, review stages, and data points for new active substances and biosimilars approved in 2017 using the Optimising Efficiencies in Regulatory Agencies (OpERA) tool. OpERA is an agency-focused metrics programme supporting the information needs of mature and maturing agencies, and building a culture of measurement to improve process timelines and quality. Through OpERA, CIRS collaboratively collects and assesses data characterizing review processes [3, 4]. Data from the year 2017 established the baseline OpERA findings and highlighted an approval process with many review cycles, long pick-up times between dossier receipt and the initiation of scientific assessment, and delays in applicants responding to deficiency questions [4]. NPRA proposed three key strategies to address these weaknesses: (i) a maximum of five review cycles when communicating with applicants on the list of questions; (ii) a target of 100 days between file acceptance and the start scientific assessment; and (iii) a requirement for applicants to provide responses within 6 months [4]. The agency has shifted its focus within risk-based evaluations, assessing not only what is locally critical, but also other risks such as active pharmaceutical ingredient contamination, for a more comprehensive evaluation. Additionally, the agency has explored what can be leveraged from stringent regulatory authorities within a reliance model to improve efficiency and reduce duplication of work [5].

The speed of marketing authorization approval at NPRA has long been recognized as a challenge, particularly given limited regulatory resources and growing submission volumes. In 2017, an initial analysis of NPRA’s regulatory performance using CIRS’ OpERA methodology identified several improvement measures [4]. However, it remains unclear whether these efforts resulted in meaningful improvements to regulatory timelines. To address this question, this study evaluates the impact of these measures by analyzing data from the year 2019. Its objectives are twofold: (1) to provide an overview of the current review process and regulatory pathways for newly approved active substances, including biosimilars, using CIRS’ standardized reporting approach; and (2) to compare time allocation in the 2019 approval process with that of 2017, utilizing CIRS’ OpERA metrics methodology, to assess whether the improvements implemented have enhanced regulatory efficiency.

## Methods

This study consists of two parts: (1) a Country Report identifying key components of the review, including registration processes, practices, and the review models; (2) a metrics tool tracking regulatory performance and measuring review timelines, assessing key granular milestones, and capturing the time spent by NPRA and the applicant.

### Country Report Using CIRS Standardized Reporting Approach

The Country Questionnaire is a Microsoft Word-based tool, completed by National Regulatory Authorities (NRAs) with guidance from CIRS. It details: (1) the organization of the NRA; (2) the types of review models used to assess medicines; (3) key milestones in the review process; (4) elements of Good Review Practices in place; and (5) quality decision-making practices used to make regulatory decisions [6]. The objectives are to accurately define the NRA’s processes and practices, provide context to enable interpretation of quantitative metrics, enable global comparisons to similar NRAs open to sharing their profile [7, 8], encourage transparent publication of information related to the NRA, and promote alignment with best practice. CIRS then develops the Country Report, using a standardized approach to identify the key components of the review, including characteristics that may impact regulatory performance and practices that bring quality to the registration processes. To support development of a second Country Report, NPRA completed the Questionnaire in 2022, focusing on new medicines registration. CIRS reviewed this and queried missing data. A draft Country Report was prepared, building upon the most recent iteration developed in 2008, and was presented and discussed with NPRA before finalization.

### OpERA Toolkit

In 2009, CIRS developed a standardized reporting approach to identify key characteristics impacting regulatory performance. The CIRS OpERA toolkit is comprised of seven key tools focused on evaluating the regulatory review process and practices, implementing a structured approach to benefit-risk, and establishing good review and decision-making practices [6].

### Metrics Methodology

The OpERA metrics methodology allows comparisons within and across regulatory agencies, as it is based on the collection of specific defined milestones that can identify where time is spent in the approval process by breaking down the key agency review stages (i.e., validation, scientific assessment, applicant and agency time, and time from the end of scientific assessment to marketing approval) through the collection of specific data points, [6]. The milestone dates set out as data points can be found in Supplementary Table 1 and were collected for each application. Additionally, qualitative data were requested for each product to characterize the application. These data included applicant name; compound type (new chemical entity, biological, or vaccine); generic name or compound code; whether the compound was a WHO pre-qualified generic or vaccine; trade name; review type; (verification, abridged, or full); therapeutic class (identified by ATC code), and whether it was a priority review. The metrics data collection for this study mirrored the data collection undertaken for NPRA approvals in 2017 [4] to enable comparisons.

### Collection and Utilization of Metrics Data from NPRA

NPRA provided data, using a CIRS-supplied Microsoft Excel spreadsheet, for NASs consisting of new chemical entities and biologics approved between 1st January 2019 and 31st December 2019. CIRS validated the integrity of milestone dates provided and evaluated missing data. The dataset for the year 2017 was sourced directly from the previously published analysis [4]. A draft analysis was prepared and presented by CIRS to NPRA to confirm a shared understanding of the dataset.

### Data Collection Timeline

Data collection was initiated in July 2020 for both Country Report and metrics, with the focus for metrics on approvals in 2019, in order to assess the outcomes of the changes implemented following the study of approvals for the year 2017. Collection, review, validation, and reporting of the data were undertaken during 2022, with finalization of the reports in November 2022. A meeting was held with NPRA in January 2023 to review the key findings.

### Metrics Analysis: Review stages and Data Points

In order to evaluate the impact of the changes implemented by NPRA since 2018, following the OPERA analysis in 2017, the three main stages of review were analyzed for NAS approved in 2019, which include: (i) time from receipt of the dossier to start of scientific assessment; (ii) scientific assessment time (including both agency assessment and applicant response time); and (iii) authorization time. These data were derived from the collection of key milestones dates for each NAS (see Supplementary Table 1), which were then analyzed statistically using medians and percentiles (specifically the 5th, 25th, 75th, and 95th percentiles) to better understand variance around the median.

### Ethics Approval and Consent

This article does not contain any studies with human or animal subjects performed by any of the authors, and informed consent is not required.

## Results

Summary results from the Country Report are presented here relating to 1—Organization of the authority; 2—Good Review Practices: Elements of Good Review Practices in place at NPRA and Quality Decision-Making Practices used to make regulatory decisions. This is followed by description of types of review models/registration pathways, the review process, key milestones in the review process, and metrics measuring the performance for approvals in 2019.

### Country Report

Organization of NPRA: NPRA is an authority that operates within the administrative structure of the Health Ministry and regulates medicinal products for human use and for veterinary use. NPRA is funded entirely by the government and currently employs approximately 500 staff members, including 101 reviewers dedicated to evaluating applications for marketing authorizations or product licenses for medicines. Of these reviewers, 33 specialize in assessing new chemical entities and biologics. The professional background of these reviewers was primarily pharmacy. NPRA’s scope of activities includes marketing authorizations/product licenses; clinical trial authorization; post-marketing surveillance; laboratory analysis of samples and site inspections/visits, GMP inspections, and the licensing of premises.

Elements of Good Review Practices (GRevP), which are key to building quality into the regulatory process, are summarized in Table 1. The agency has identified three key measures for introducing quality initiatives: improving efficiency, ensuring consistency, and enhancing stakeholder satisfaction. GRevP have been applied by the agency in the review practice, through guidelines, internal standard operating procedures (for reviewers and advisory committee members) along with evaluation templates and checklists. NPRA’s internal quality policy, ISO 9001:2015 certified since July 2017, and its dedicated quality department reflect its commitment to international standards and delivering quality products and services. In addition, the Country Report identified other quality measures instigated by NPRA, including transparency and communication, emphasizing the agency's high priority on maintaining openness and transparency in its relationships with the public, professionals, and industry. Examples of this include providing details of technical staff to contact, official guidelines to assist applicants, as well as providing the ability to track the progress of applications. The agency identified three key incentives for allocating resources to activities that enhance regulatory system transparency: ensuring safety measures; improving staff morale and performance, and strengthening confidence in the system.

The agency also supports continuous improvement through external and internal audits, review of both assessor and applicant feedback, and utilization of an internal tracking system to monitor consistency, timeliness, efficiency, and accuracy of the review. The agency conducts on-the-job training and provides internal and external training programmes for assessors, which are sometimes undertaken in collaboration with other agencies. Several training programmes include examinations and successfully completing these courses are mandatory for professional advancement. Agency staff also have opportunities to attend international conferences and may receive the Ministry of Health’s support for pursuing a postgraduate degree. The agency had not implemented placements or secondments to other regulatory authorities as a training method for assessors.

**Table 1:** Quality in the review process

✓	Quality management: The agency puts a high priority on building quality into its processes and has measures in place to monitor and improve quality.
✓	Consistency: A priority reason for implementing quality measures is to ensure consistency, to increase efficiency, and to achieve stakeholder satisfaction.
✓	SOPs: Standard operating procedures have been implemented as part of the quality measures adopted by the agency.
✓	Assessment templates: These are used to standardize the content and format of written reports.
✓	Transparency: The agency assigns a high priority to being open and transparent in its relationships with the public, professions, and industry.
✓	Monitoring application progress: An electronic tracking system is in place.

Quality decision-making practices: NPRA has a formally defined and codified framework in place that forms the basis of the decision to approve or reject a Marketing Authorization Application (MAA). Key measures implemented by NPRA include having a structured approach; assigning clear roles and responsibilities (decision makers, advisors, information providers); evaluating both internal and external influences or biases; examining alternative solutions; and considering uncertainty. NPRA also re-evaluate as new information becomes available for an MAA, ensure transparency of the process, provision of a record trail, and effectively communicate the basis of the decision.

**Fig. 1:**
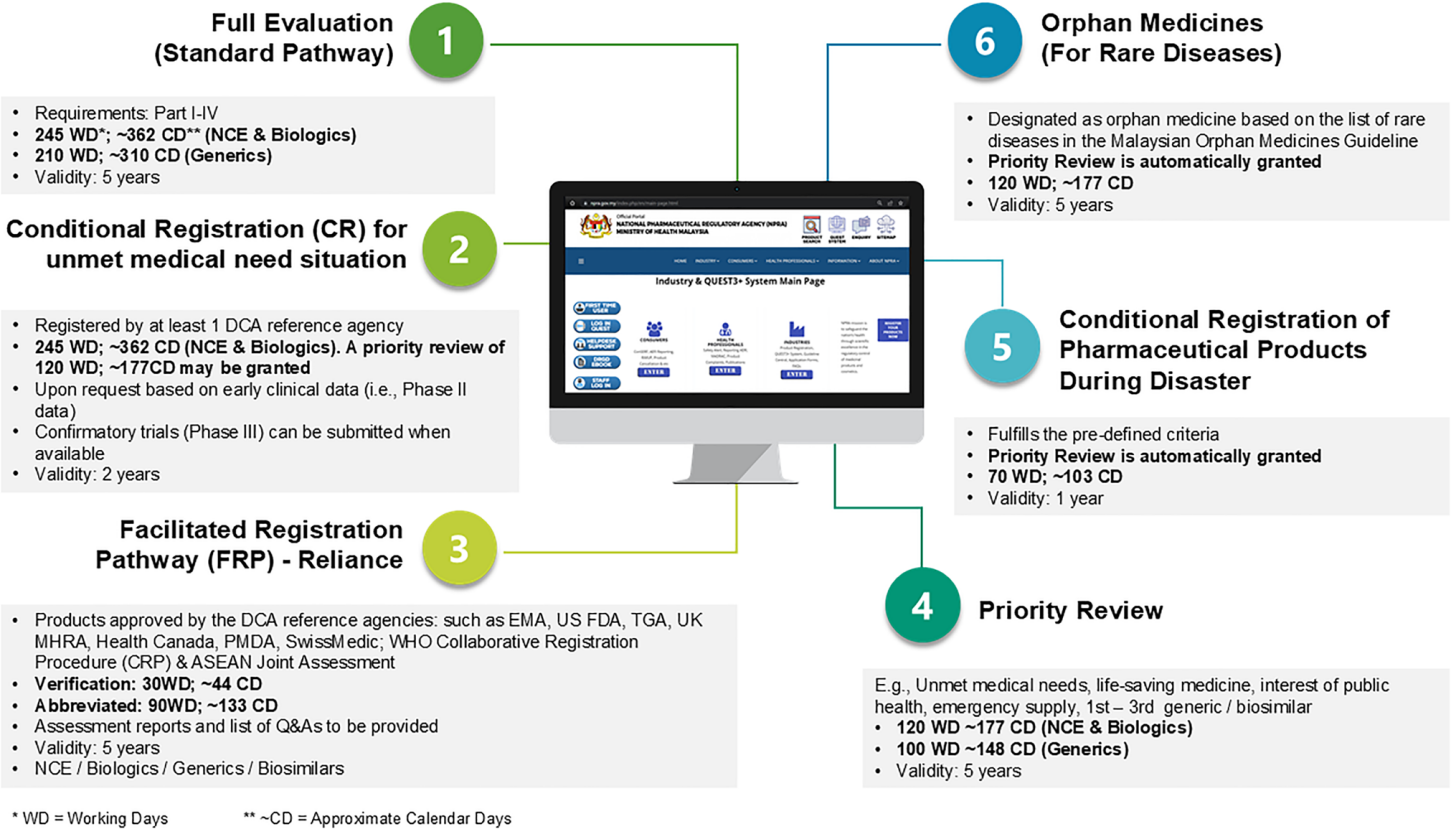
Registration pathways

The registration pathways in Malaysia: Like other NRAs, NPRA has introduced alternative registration pathways to expedite approvals and improve patient access. Fig. 1 illustrates the alternative registration pathways, including their criteria and timelines. Apart from standard pathway, NPRA also allows certain promising new medicines, including vaccines, to reach patients with unmet medical needs earlier (via the conditional registration pathway) while ensuring measures are in place to manage the risks inherent in additional data still being required [7]. NPRA has adopted the principle of reliance through Facilitated Registration Pathways (FRPs), enabling an expedited registration process by relying on reference agencies’ assessment reports for products seeking approval in Malaysia. Other abbreviated pathways with shorter timelines are Orphan Medicines, Conditional Registration of Pharmaceutical Products During Disaster, and Priority Review; for which pre-defined criteria must first be met. The majority of NASs approved in 2019 went through the standard approval pathway.

**Fig. 2:**
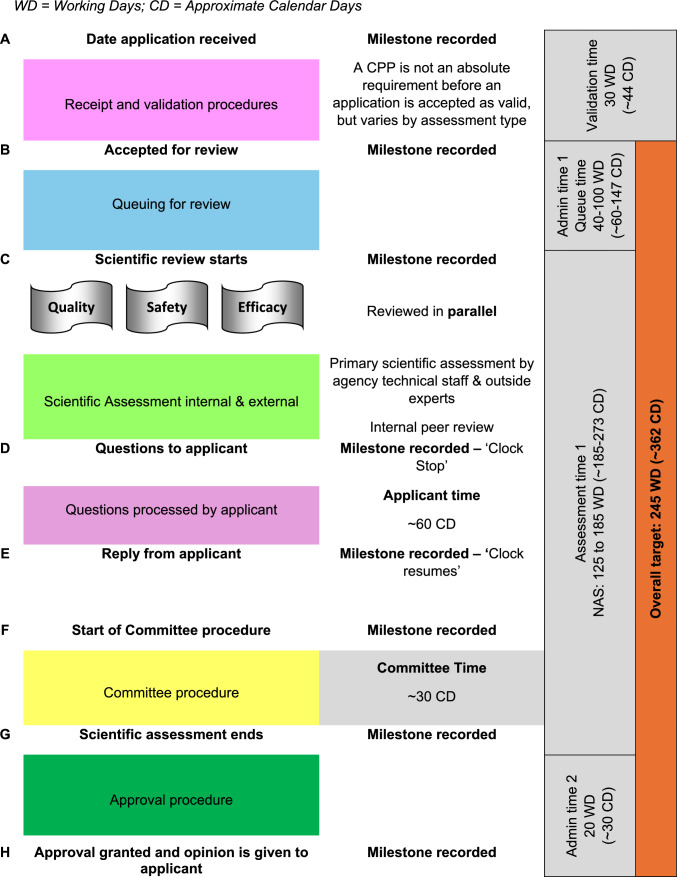
The review process at NPRA. *WD* Working Days, *CD* Approximate Calendar Days

Review process: The review process is described in Fig. 2 (steps A to H) and key aspects of the marketing authorization process are summarized in Table 2. Upon submission of an ASEAN Common Technical Dossier (ACTD) via the online system, the dossier is validated to ensure completeness. The application is accepted if all essential documents are provided, and scientific regulatory review ensues to evaluate the product's quality, efficacy, and safety. External inputs are sought from appointed experts or clinical specialists in the relevant field for NASs and biosimilars. After initial review, the assessor corresponds with the applicant, providing a list of questions to request further information or documents. Once the additional data is deemed satisfactory, the final evaluation report is presented at NPRA’s product evaluation committee meeting, followed by presentation to the Drug Control Authority (DCA) for final marketing authorization decision.

**Table 2:** Key characteristics of NPRA marketing authorization process

✓	Certificate of a Pharmaceutical Product (CPP): Requirements for submitting a CPP at the point of application submission are flexible
✗	Medical staff: More than 25% of the agency review staff are medically qualified
✓	Review times: The agency sets targets for the time it spends on the scientific assessment of NAS applications
✓	Approval times: The agency has a target for the overall time for the review and approval of an application
✓	Questions to applicants are batched at fixed points in the review procedure
✓	Applicant response time: Recording procedures allow the applicant response time to be measured and differentiated in the overall processing time
✓	Priority reviews: The agency recognizes medical urgency as a criterion for accelerating the review and approval process for qualifying products
✓	Parallel processing: The different sections of technical data (Quality, Safety, Efficacy) are reviewed in parallel rather than sequentially
✓	Price negotiation: Discussion of pricing is separate from the technical review and does not hold up the approval of products
✓	Sample analysis: The focus is on checking quality in the marketplace and requirements for analytical work do not hold up the marketing authorization

### Metrics Results

Twenty-four NASs were approved by NPRA in 2019, of which 10 (42%) were new chemical entities and 14 (58%) were biologics (including 1 vaccine). The included products were also categorized based on their assessment route: Path I (priority review), Path II (standard review), or Path III (Facilitated Registration Pathway). Of products approved in 2019, 1 (4%) was assigned to Path I, while 3 (12%) were designated Path III (Table 3).

**Table 3:** Characteristics of 24 NAS approved by NPRA in 2019

	NASs approved in 2019
Total Number of NASs	24
Compound types	
New chemical entities	10
Biologics (of which are vaccines)	13 (1)
Assessment route	
Path I (priority review pathway—full review)	1
Path II (standard review pathway—full review)	20
Path III (facilitated registration pathway—abbreviated/verification)	3

The therapeutic areas of the products approved vary, with anti-cancer, immunomodulatory, and anti-diabetic products representing a combined proportion of 50%.

### Overall Approval Times

The median time for overall approval, from the date application received (Step A) to approval granted (Step H) (see Fig. 2), including both applicant and agency time, for the 24 NAS was 399 calendar days. The inter quartile range (IQR) was 275 to 472 days (25th to 75th percentile). NPRA’s review timelines for new chemical entities and biologics are 245 working days (~362 calendar days) for standard review and 120 working days (~177 calendar days) for priority review (Fig. 1). However, these targets are for agency time only, rather than overall approval time.

### Breakdown of Time Spent in the Approval Process

**Fig. 3:**
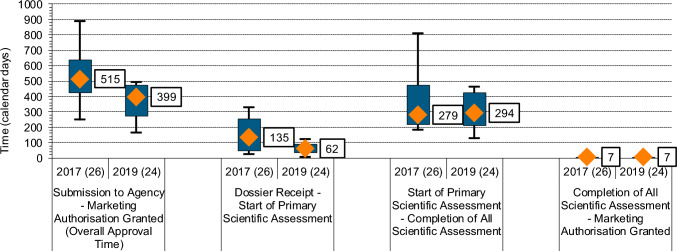
The three main components of the approval process for 24 NAS approved by NPRA in the year 2019 compared to 2017; *n* number of applications

The approval process is divided into three key stages: (i) time from receipt of the dossier to the start of scientific assessment; (ii) scientific assessment time; and (iii) authorization time. The median time for all stages is shown in Fig. 3. Comparing the three main stages of NPRA approval process for medicines approved in 2019, the agency spends most of its time in scientific assessment compared to the other two stages. The median time for scientific assessment, which includes both agency and applicant time, was 294 calendar days with an IQR range of 209 to 421 calendar days (25th to 75th percentile). Meanwhile, the median time from dossier receipt to the start of scientific assessment, was 62 calendar days (an IQR of 35 to 88 calendar days), whereas the median time for the authorization stage, from scientific assessment completion to marketing authorization, was 7 calendar days, matching the median for the year 2017 (Fig. 3).

Comparing the results for the year 2019 to those for 2017 (Fig. 3) for the same milestones showed an overall reduction in approval time (399 vs 515 calendar days) as well as the time from receipt of the dossier to the start of scientific assessment (135 vs 62 calendar days). However, the median scientific assessment time increased slightly by 15 calendar days (294 vs 279 calendar days).

### Time from Dossier Receipt to Start of Scientific Assessment

**Fig. 4:**
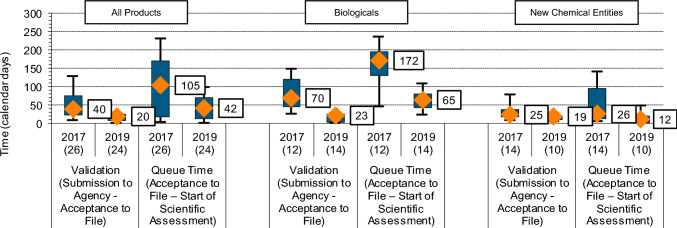
Time from dossier receipt to start of scientific assessment for the years 2019 and 2017 by product type, validation (submission to agency to acceptance to file) and queue time (acceptance to file to start of primary assessment); *n* number of applications

The two components of this stage are: (i) validation time (from submission made to agency’s acceptance of the file), and (ii) queue time (from agency acceptance to the start of scientific assessment). The overall median of the two components in 2019 was 20 calendar days (IQR of 8 to 25 calendar days) and 42 calendar days (IQR of 13 to 71 calendar days) for validation time and queue time respectively (Fig. 4). When compared to the year 2017, overall improvement was observed in validation time and queue time. The reduction in time for the year 2019 was most notable for biologics. While new chemical entities also showed reductions in the validation time and the queue time, the decreases were less substantial (Fig. 4).

### Scientific Assessment Including Agency and Applicant Time

Overall scientific assessment time was observed to have increased slightly for the year 2019 compared to 2017 (Fig. 3). A breakdown by product type revealed that median scientific assessment time (which includes agency and applicant time) for biologics increased by 19 calendar days (IQR: 2019, 204–319 calendar days; 2017, 212–255 calendar days), while the median scientific assessment time for new chemical entities decreased by 44 calendar days (IQR: 2019, 295–434 calendar days; 2017, 305–641 calendar days).

Meanwhile, median agency time in 2019 vs 2017 increased for both biologics (165 vs 116 calendar days) and new chemical entities (234 vs 211 calendar days), respectively. Reduction in applicant time was noted for both biologics (95 vs 101 calendar days) and new chemical entities (179 vs 229 calendar days) respectively. For new chemical entities, a drop in IQR from 360 calendar days in 2017 to 110 calendar days in 2019 was also noted.

**Fig. 5:**
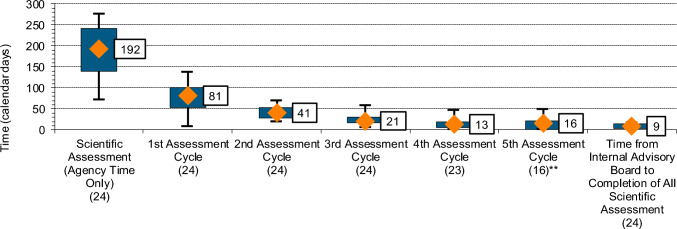
Agency and applicant scientific assessment time for 24 NASs approved by NPRA in 2019. *n* number of applications

Agency scientific assessment time was broken down further into primary scientific assessment, the time taken for the agency to review the dossier prior to sending out the first set of questions and the subsequent time for applicant’s responses. This process may be repeated, resulting in several review cycles. The median primary scientific assessment time for the 24 NASs approved in 2019 was 81 calendar days (Fig. 5), equivalent to approximately 42% of total agency scientific assessment. Compared to the year 2017, this had increased by 31 calendar days. In 2017, the median primary scientific assessment time of 50 calendar days represented approximately 29% of the total agency scientific assessment time [4].

Median total applicant time to respond to questions was 108 calendar days for the NASs approved in 2019. This compares with a median of 131 calendar days for total applicant time in 2017 [4]. Applicants took a median of 44 calendar days to respond to questions raised by the agency following the primary scientific assessment (41% of total applicant time). In 2017 this was 41 calendar days (29% of total applicant response time). For this cohort of 24 NASs, completion of scientific assessment took 3 cycles for 1 NAS (4.2%), 4 cycles for 7 NASs (29.2%), and 5 cycles for the remaining 16 NASs (66.7%). In 2017, 2 cycles were taken for 1 NAS (3.8%), 3 cycles for 3 NASs (11.5%), 4 cycles for 9 NASs (34.6%), 5 cycles for 8 NASs (30.8%), and 6 cycles for 5 NASs (19.2%) [4].

## Discussion

Understanding the review process by documenting and measuring it is key to ensuring that quality is built into the process and that reasonable timelines are involved [9]. Through the Country Report, it was identified that GRevP has been implemented in NPRA’s review process. The agency has most of the key quality measures, as well as key and identifiable quality decision making processes [10] in place, to facilitate the regulatory review of medicines. NPRA recognizes the importance of training, offering both internal and external opportunities to ensure that reviewers receive comprehensive and proper training.

Transparency of the process is also a priority and is instrumental to building trust with the agency’s stakeholders, including industry and patients. The multiple registration pathways provided by NPRA enhance accessibility and efficiency in the marketing approval process for medicines. In 2017, NPRA utilised two main pathways: standard review (Path II) and priority review (Path I), with eligibility based on public health need and prior registration in reference countries [4]. There are now flexible routes tailored to various product types that fulfil the set eligibility criteria. This approach reduces time to market for medicines and improves regulatory efficiency by allowing NPRA to manage and prioritize applications based on complexity and urgency. By adapting regulatory processes to specific product needs, these pathways support innovation and ensure a more effective and responsive system for the pharmaceutical industry and public health. Since the implementation of this FRP in April 2019 [11], only three of the approvals in that year were reviewed through this pathway. Utilizing metrics provides an evidence-based approach to identifying areas for improvement and assessing the impact of any changes on the regulatory landscape and agency practices. For NPRA, applying the CIRS OpERA methodology enabled evaluation of the changes implemented following analysis of data from the year 2017 on new medicine approvals. Using the same methodology, NPRA assessed the impact of these changes on products approved in 2019. This was achieved by comparing the approval metrics for the year 2017 to those for 2019, providing insights into the effectiveness of the adjustments.

### Total Approval Time

A decrease in median and a slight reduction in the IQR of the total approval time was seen for products approved in 2019 compared to those approved in 2017. Indeed, the variability reduced from 208 calendar days in 2017 to 197 calendar days in 2019, but a major shift in total approval time was also observed as the 75th percentile in 2019 (472 calendar days) was similar to the 25th percentile in 2017 (427 calendar days), showing improvement across all applications approved. The reasons for overall reduction in approval time in 2019 were explored and found to be due to the changes made within the review process by NPRA. These included reducing the time to validate the dossier and pick-up time following validation by the assessor, reducing the number of review cycles, and limiting overall applicant time [12].

### Time from Dossier Receipt to Start of Scientific Assessment

The analysis of approvals for the year 2017 identified that the validation and queue time, before pick-up by a reviewer, was a key area causing delays in the overall approval time and was also an area which showed difference between the new chemical entity and biological review divisions. In 2017, there were no maximum limits on the number of review cycles or applicant response times, and no target timeline for initiating scientific assessment once a dossier had been accepted for review [4]. The agency made several proactive changes in 2018 with regards to these parts of the review, which included instigating a target start for scientific assessment of 100 days after file acceptance, a maximum of 5 review cycles, and applicant response time limited to 6 months [4]. Work practices were also reengineered to improve efficiency by streamlining the processes. The approvals for the year 2017 had a median time for the dossier to be picked up for review following submission of 135 days, with a wide variation of 46 to 254 days (25th to 75th percentile); a delta of 208 days. The time to pick-up the dossier for assessment was dramatically reduced to a median of 62 days for the cohort of compounds approved in 2019, and the variation in pick-up time ranged from 35 to 88 days (25th to 75th percentile); a delta of 53 days. This reduction in pick-up time was observed for both biologics and new chemical entities and showed that the agency was within the target time set, suggesting the new processes and practices had a positive impact.

### Scientific Assessment Time Including Agency and Applicant Time

In 2017, the median time spent by the agency on scientific assessment was 166 days (with the median applicant time of 131 days) and some products involved up to six cycles of correspondences before approval. Notable in the approvals for the year 2017 was a large variation in applicant time, with the IQR being 94 to 238 days. NPRA introduced steps which limit applicants to up to five rounds of correspondence and with a maximum of applicant time of 6 months; failure to adhere to this timeframe would result in application rejection. This mechanism helps control applicant time, although applicants may request extensions. Approvals for the year 2019 showed a minor increase in median scientific assessment time, primarily due to more agency time spent on the review, particularly during the primary scientific assessment. In 2019, 42% of the total agency assessment time was spent on this stage, compared to 29% in 2017. Despite this, most approvals required 5 cycles, with only 8 of 24 approvals requiring fewer cycles. Although 5 cycles is within the target, it may suggest reviewers were raising more questions on applicants’ responses, or that applicants were not fully answering the agency’s questions, necessitating multiple cycles. Achieving fewer review cycles can help reduce the resources needed by the agency and increase the efficiency of the process. That said, the total median applicant time reduced from 131 days in 2017 to 108 days in 2019, indicating that the mechanisms NPRA put in place may have helped reduce applicant time. NPRA believes that a maximum of five review cycles is essential to ensure thorough product evaluation and compliance with regulatory standards [4]. This extensive review process is supported by frequent stakeholder engagement and technical assistance, including pre-submission meetings [13, 14], which help clarify submission procedures and regulatory requirements. Such ongoing interaction enhances regulatory compliance and responsiveness, contributing to a more efficient and effective review process.

The study’s findings are constrained by the utilization of data primarily from the year 2019, which may not fully reflect current conditions. The study was planned to start in 2020, to evaluate changes implemented in 2018. However, the COVID-19 pandemic delayed data collection, review, validation, and reporting until the year 2022. Data for the years 2020–2022 were excluded due to concerns they may not represent normal conditions, given pandemic-related changes in evaluation processes and priorities. Nonetheless, regulatory review practices are mostly unchanged from the year 2019, which suggests the study’s findings remain relevant. However, the exclusion of recent data and the pandemic’s impact on evaluation procedures may limit the results’ overall applicability and timeliness.

Inferential statistical tests were not applied as the study lacked sufficient power to detect moderate effects, given the small sample sizes for 2017 and 2019 (n = 26 and n = 24, respectively). A sample size of approximately 64 approvals per year would be required to support robust inferential statistical testing (e.g., Mann-Whitney U test). Future studies with larger sample sizes may allow the use of inferential statistical testing to determine significance.

## Summary and Conclusion

The study findings indicate that the changes implemented in 2018 had a favorable impact on reducing overall approval times, as evidenced by the comparison of products approved in 2017 and 2019. Moving forward, as a regulatory authority, NPRA’s commitment to regulatory excellence is underscored by several key strategies. Firstly, prioritizing collaboration and mutual trust with other NRAs, which is crucial for building capacity, achieving regulatory convergence, and harmonizing efforts across jurisdictions. Additionally, the ASEAN Joint Assessment initiative [15] has improved collaboration among member states, deepened understanding of reliance mechanisms, enhanced pharmaceutical assessment capacities through shared experiences, and facilitated simultaneous market access and timely patient care across ASEAN countries. By working together, a more cohesive regulatory environment can be developed. Secondly, adopting new approaches in regulatory practice such as reliance [5], enabling NPRA to use resources more efficiently and minimize duplication of efforts. Current regulatory processes are regularly reviewed to eliminate less impactful ones, streamlining operations and improving overall efficiency. This highlights the importance of agencies consistently measuring key components of the review process. Thirdly, the revision of guidelines and upgrading of systems are pivotal in supporting the implementation of the agency regulatory framework. Regular updates and revisions ensure that the practices align with global standards and remain relevant in a rapidly evolving regulatory landscape. This holistic approach will enhance NPRA’s ability to effectively manage and oversee regulatory activities while promoting consistency and efficiency.

## Supplementary Information

Below is the link to the electronic supplementary material.

Supplementary file1 (DOCX 33 KB)

## Data Availability

No datasets were generated or analysed during the current study.
