# Supplementary material for: Assessing the Malaysian Regulatory Process for Medicinal Product Approval: An OpERA Methodology and Standardized Reporting Approach
Source: Ther Innov Regul Sci. 2025 Jul 25;59(6):1452–62. doi: 10.1007/s43441-025-00846-3 (PMC12579699; doi:10.1007/s43441-025-00846-3)
Supplement: Supplementary file 1 — Supplementary file1 (DOCX 33 KB) [file 43441_2025_846_MOESM1_ESM.docx]

**Supplementary Table 1. Metric datasets collected through the OpERA Programme.**

| **Key Milestone Date** | **Time** | **Time Period Measurement** |
| --- | --- | --- |
| 1a. Receipt of the dossier | Dossier validation time | *Dossier validation*: The time between the date stamped on receipt of the dossier and the date of sending the acceptance (or refusal)-to-file letter. |
| 1b. Acceptance to file | Time to acceptance of the dossier | *Queue time*: Time between accepting the dossier for review and starting the review. |
| 2a. Start of primary scientific assessment | Primary scientific assessment | *Scientific assessment time*: Time spent between the start date of the scientific assessment to the date of completion of all scientific assessments.  *Agency scientific assessment time*: Amount of time spent between the start of the scientific assessment to the date of the completion of all scientific assessments, minus the time the applicant needs to prepare responses to questions or the time for any additional information to be provided by the applicant.  *Applicant time*: The time during which the review timeline clock is stopped during the review while the authority awaits additional data requested from the applicant. |
| 2b. Completion of primary scientific assessment |  |  |
| 3a. Primary assessment deficiency letter sent to applicant (if applicable) | Clock stop / Applicant time |  |
| 3b. Response from applicant (if applicable) |  |  |
| 4. Secondary assessment following deficiency letter response (if applicable) | Secondary scientific assessment time |  |
| 5. Succeeding Advisory Committee review (if applicable) | Advisory Committee time | *Authorization time*: The time from completion of all scientific assessments to the authorization/license date that allows legal marketing. |
| 6. Completion of Scientific Assessment | When all assessment activities are completed |  |
| 7. Marketing Authorization granted/rejected | Date granted/rejected | *Overall approval time*: The time between the date stamped on receipt of the dossier when received by the authority and the date on the document (authorization/license) that allows legal marketing. |
